# Effects of Replacing Fish Meal with Enzymatic Cottonseed Protein on the Growth Performance, Immunity, Antioxidation, and Intestinal Health of Chinese Soft-Shelled Turtle (*Pelodiscus sinensis*)

**DOI:** 10.1155/2023/6628805

**Published:** 2023-05-24

**Authors:** Zongsheng Qiu, Jiantao Zhao, Dazhang Xie, Clement R. de Cruz, Jianhua Zhao, Hong Xu, Qiyou Xu

**Affiliations:** ^1^School of Life Science, Huzhou University, Huzhou 313000, China; ^2^Zhejiang Provincial Key Laboratory of Aquatic Resources Conservation and Development, Huzhou University, 759 Erhuan Road (E), Huzhou 313000, China; ^3^Zhejiang Jindadi Biotechnology Co., Ltd., Shaoxing 311800, China; ^4^Department of Aquaculture, Faculty of Agriculture, Universiti Putra Malaysia, 43400 Serdang, Selangor, Malaysia

## Abstract

The dietary effects of replacing fish meal with enzymatic cottonseed protein (ECP) on the growth performance, immunity, antioxidant, and intestinal health of Chinese soft-shelled turtles have not been explored. An eight-week feeding trial was conducted with a quadruplicated group of turtles (3.44 ± 0.01 g) that were randomly assigned to 16 cages (0.6 m × 0.6 m × 0.6 m) with 30 turtles that were stocked in each cage. Four dietary groups were fed with diets supplemented with 0, 2%, 4%, and 6% (ECP0 group (control group), ECP2 group, ECP4 group, ECP6 group) of enzymatic cottonseed protein replacing fishmeal. The present study illustrated that the final weight and WG in the ECP2 and ECP4 groups were significantly increased (*P* < 0.05) compared with the control group. The ECP2, ECP4, and ECP6 groups significantly reduced the feed coefficient (*P* < 0.05) and significantly increased the SGR (*P* < 0.05). The serum TP and ALB of the ECP4 group were significantly increased (*P* < 0.05). The ECP2, ECP4, and ECP6 groups significantly increased the activity of intestinal pepsin (*P* < 0.05), and the activity of intestinal lipase of the EPC4 group was significantly increased (*P* < 0.05). The intestinal villus height of the EPC4 group and EPC6 group, the villus width of the EPC2 group and EPC4 group, and the intestinal muscle thickness of the EPC4 group were significantly increased (*P* < 0.05). At the same time, replacing fishmeal with enzymatic cottonseed protein also affected the intestinal inflammation-related genes compared with the control group. Besides that, the expression of the *IL-10* gene in the experimental group was significantly upregulated (*P* < 0.05). Nevertheless, the expression of *TNF-α* and *IL-8* genes in the ECP2 group and *TNF-α* and *IL-1β* genes in the ECP4 group was significantly downregulated (*P* < 0.05). In summary, replacing fish meal with enzymatic cottonseed protein positively affects the growth, immunity, and intestinal health of Chinese soft-shelled turtles. The appropriate proportion of enzymatic cottonseed protein to replace fish meal in turtle feed is 4%.

## 1. Introduction

In the traditional aquaculture, the feed usually accounts for 60%-70% of the total aquaculture cost [1]. As a major feed cost component, protein is the most important factor affecting aquatic animals' growth performance and feed cost [2]. With the development of intensive aquaculture, the main protein resource fish meal is in short supply [3], and the price is rising. Excessive use of fish meal in the feed will increase feed cost, so the aquaculture industry is looking for new protein sources to replace the fish meal [4]. Plant protein sources have abundant resources, stable supply, and safety advantages, which have received greater attention from researchers in animal nutrition and feed development niches for the past decades [5, 6]. However, there are still certain limitations in applying plant protein in feed. Excessive plant protein will increase the oxidative stress of aquatic animals and cause body damage [7]. The factors affecting the utilization of plant protein sources by aquatic animals mainly include different feeding habits of aquatic animals, different adaptability to plant protein sources at different stages of growth and development [8], unbalanced amino acid composition [9], many kinds of antinutritional factors [10], and poor palatability [11]. Studies have confirmed that proteins in animals are absorbed in the form of small peptides and amino acids, and small peptides are absorbed faster and can avoid competition with amino acid absorption [12].

Cottonseed protein removes free gossypol while protecting protein and amino acids from damage to the maximum extent [13]. The content and quality of protein are significantly higher than those of cottonseed meal pressed at high temperature, and the amino acid composition is more balanced. The contents of arginine, histidine, and phenylalanine in cottonseed protein were equal to or higher than those in fish meal, but other amino acid levels were still lower than those in fish meal [14]. Enzymatic hydrolysis uses specific enzymes to carry out corresponding chemical reactions to remove some particular structures. The macromolecular protein can be decomposed into a variety of small peptides, peptide substances, and amino acids through the appropriate protease to hydrolyze the peptide bonds in the protein [15]. Enzymatic hydrolysis can increase the content of water-soluble protein, total amino acid, and peptide components of cottonseed protein [16]. In addition, a variety of active peptides can be produced after enzymatic hydrolysis, such as antioxidant peptides and antimicrobial peptides [17, 18]. A large number of studies have shown that enzymatic protein products in diets can relieve the digestive burden of animals, increase food intake and feed utilization [19, 20], promote the development of intestinal structure [21, 22], and enhance immunity [23, 24].

Chinese soft-shelled turtle (*Pelodiscus sinensis*) has many advantages, such as stable genetic traits, fast growth, strong disease resistance, high nutritional value, and good breeding economic benefits. Fish meal is the most important and expensive component in the feed of Chinese soft-shelled turtles. After undergoing enzymatic hydrolysis, cottonseed protein increases its amino acid and small peptide content. It also produces antioxidant, anti-inflammatory, and other active peptides. Cottonseed protein can be used as a new protein source in place of the fish meal due to its ability to promote intestinal development and structural stability [25, 26]. This paper intends to study the effects of replacing fish meal with enzymatic cottonseed protein on the growth performance, immunity, antioxidation, and intestinal function of Chinese soft-shelled turtles.

## 2. Materials and Methods

### 2.1. Animal Ethics

The study was conducted in accordance with the Declaration of Helsinki and approved by the medical ethics committee of Huzhou University (20220916).

### 2.2. Experimental Ingredients and Diets

The main nutritional components of the enzymatic cottonseed protein used in the experiment are 62% crude protein and 0.4% crude lipid. The formula was designed according to the nutritional requirements of Chinese soft-shelled turtle [27–30]. The experimental feed was prepared with fish meal as the protein source, wheat meal as the sugar source, and soybean oil as the fat source. Four experimental diets were prepared by replacing the same amount of fish meal with 0, 2%, 4%, and 6% (ECP0 group (control group), ECP2 group, ECP4 group, and ECP6 group) enzymatic cottonseed protein in the basal diet. The formulas and nutritional levels of each experimental feed are shown in Table 1. All ingredients are finely ground and thoroughly mixed through a blender. The 2.2 mm expanded particles were made by an expanded granulator and dried by an air dryer.

### 2.3. The Feeding Trial

The feeding trial was carried out for eight weeks in the greenhouse breeding system of Zhuji Jindadi Agricultural Co., Ltd., Shaoxing City, Zhejiang Province. The turtles hatched at the same time were temporarily raised. A total of 480 Chinese soft-shelled turtles with an average weight of 3.44 ± 0.01 g were randomly distributed to 16 cages (0.6 m × 0.6 m × 0.6 m). Each dietary group had four replicates, and 30 turtles were stock in each replicate cage. The turtles were fed three times a day (6 : 00, 12 : 00, 18 : 00 h), and the feeding amount was set at 4% of the body weight. During the experiment, the water temperature was maintained at 30 ± 1°C, the dissolved oxygen was kept at more than 5 mg/L, the ammonia nitrogen was less than 0.8 mg/L, and the nitrite was less than 0.05 mg/L [31].

The turtles were weighed at the start (initial body weight) and end (final body weight) of the 56-day feeding experiment. The survival rate, weight gain, and feed coefficient of each treatment group were recorded and analyzed. These parameters were calculated as follows:

(1) $$\begin{array}{c} \text{Survival rate }\left(\%\right)=\left(\frac{N_{f}}{N_{i}}\right)\times 100,\\ \text{Weight gain }\left(\text{WG},\%\right)=\frac{\left(W_{f}-W_{i}\right)}{W_{i}}\times 100,\\ \text{SGR }\left(\text{Specific growth rate},\frac{\%}{\text{day}}\right)=\left(\frac{\left(\ln W_{f}–\ln W_{i}\right)}{\text{days}}\right)\times 100,\\ \text{Feed conversion ratio }\left(\text{FCR}\right)=\text{Feed }\frac{\text{consumed}\left(\text{g}\right)}{\left(W_{f}-W_{i}\right)}, \end{array}$$

where *W*_*f*_ and *W*_*i*_ are the initial and final body weight; *N*_*f*_ and *N*_*i*_ are the final and initial numbers of turtles.

### 2.4. Sample Collection

At the end of the feeding trial, three turtles were randomly selected from each replicate and used for whole-body composition analysis [32]. Three turtles in each group were decapitated, and blood was collected in a 2 mL centrifuge tube, placed at 4°C for 6 h, and centrifuged at 3000 rpm for 10 min to obtain serum samples. Then, the turtle was dissected, and the liver and intestine were separated. The tissue was mixed with 0.9% sodium chloride solution at a ratio of 1 : 9 (mass/volume), homogenized with a homogenizer, then centrifuged at 2500 rpm for 10 min, and the supernatant was stored in a -80°C refrigerator for subsequent analysis. The midguts were placed in Bouin's fixative solution to make intestinal histological sections.

### 2.5. Biochemical Analysis

Serum total protein (TB), albumin (ALB), globulin (GLB), lysozyme (LZM), and alkaline phosphatase (AKP) were measured. The contents of superoxide dismutase (SOD), reduced glutathione (GSH), catalase (CAT), aspartate aminotransferase (GOT), and malondialdehyde (MDA) in the liver were determined. The activities of intestinal trypsin, pepsin, lipase, and amylase were determined. The above indicators were measured and analyzed using kits produced by the Nanjing Jiancheng Institute of Bioengineering.

### 2.6. Intestinal Histological Analysis

The midgut tissue was fixed in Bouin's fixative solution for 48 hours, dehydrated with ethanol, made transparent with xylene, embedded in paraffin, sectioned, and stained with hematoxylin-eosin (HE). Next, the morphological features of the intestinal villi were observed under a light microscope, and photographs were taken. Villi height, width, and muscular layer thickness were measured with a micrometre ruler.

### 2.7. Analysis of Intestinal Gene Expression

Total RNA was extracted from intestinal tissue samples of turtles according to the method of RNA rapid extraction kit (RN28-EASYspin Plus), and cDNA was synthesized by reverse transcription kit (MonScript™ 5x RTIIII All-in-One Mix). The antibody dye quantitative PCR master mix (Mon Amp TM SYBR ® Green q PCR Mix) was used to perform RT-qPCR on the real-time quantitative PCR instrument (ABI 7500), and the specific operation steps were carried out according to the instructions. The reaction system was 20 *μ*L, including 10 *μ*L MonAmp TM SYBR ® Green qPCR Mix (Mona, China), 0.4 *μ*L forward and reverse primers (10 *μ*mol/L), 1.5 *μ*L template cDNA, 7.7 *μ*L nuclease-free water. The thermal cycle program of qRT-PCR was as follows: 95°C for 5 min, then 40 cycles at 95°C for 10 s, 60°C for 30 s, and 72°C for 30 s. The melting curve was drawn to determine the correctness of the amplification product. The 2^−ΔΔCt^ method was used to calculate the relative mRNA expression of each gene. The primer sequences of intestinal health-related genes of turtles are shown in Table 2.

### 2.8. Statistical Analysis

All statistical analyses were conducted using SPSS 18.0 for Windows. Experimental data were expressed as mean ± standard error (mean ± SE). All evaluated variables were subjected to analysis of variance (ANOVA) to determine if the levels of enzymatic cottonseed protein significantly (*P* < 0.05) affected the observed responses. In addition, to determine if the effect was linear and/or quadratic, a follow-up trend analysis using orthogonal polynomial contrasts was performed [33].

## 3. Results

### 3.1. Growth Performance and Whole-Body Composition

Compared with the control group, the FBW and WG of turtles in the ECP2 and ECP4 groups were significantly increased (*P* < 0.05). The dietary groups of ECP2, ECP4, and ECP6 significantly decreased FCR and increased SGR (*P* < 0.05) compared to the control group (ECP0). There was no significant difference in the SR among all dietary treatments (*P* > 0.05) (Table 3).

As shown in Table 4, no significant difference was found in the whole-body composition of major nutrients in all groups (*P* > 0.05).

### 3.2. Serum Biochemical Indicators

**Figure 1:**
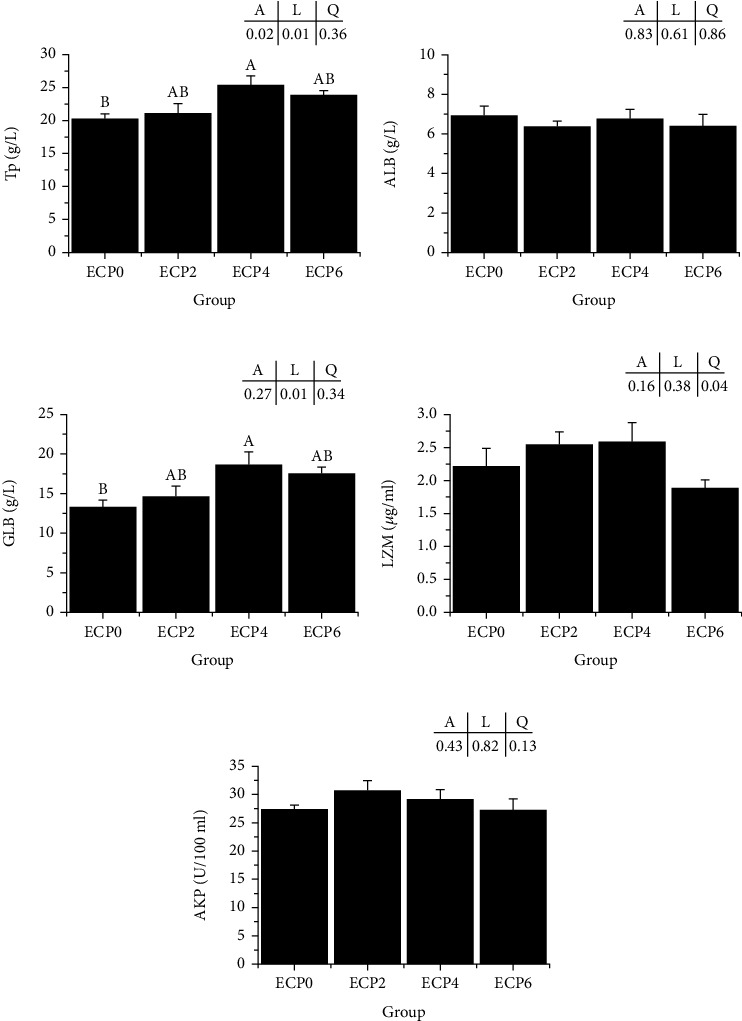
Effects of different levels of enzymatic cottonseed protein (ECP) on serum nonspecific immunity of Chinese soft-shelled turtle. (a) Tp: total protein; (b) ALB: albumin; (c) GLB: globulin; (d) LZM: lysozyme; (e) AKP: alkaline phosphatase; error bars represented the mean ± SE (*n* = 6). Different letters indicated significant differences between groups (*P* < 0.05). *A* is the variance analyzed by one-way ANOVA; *L* is the linear trend analyzed by orthogonal polynomial contrasts; *Q* is the quadratic trend analyzed by orthogonal polynomial contrasts.

As shown in Figure 1, the analysis results of the serum biochemical indicators of turtles showed that compared with the ECP0 group, the serum TP and GLB contents of ECP4 were significantly increased (*P* < 0.05). There were no significant differences in ALB, LZM, and AKP contents among the groups (*P* > 0.05).

### 3.3. Liver Antioxidant Parameters

**Figure 2:**
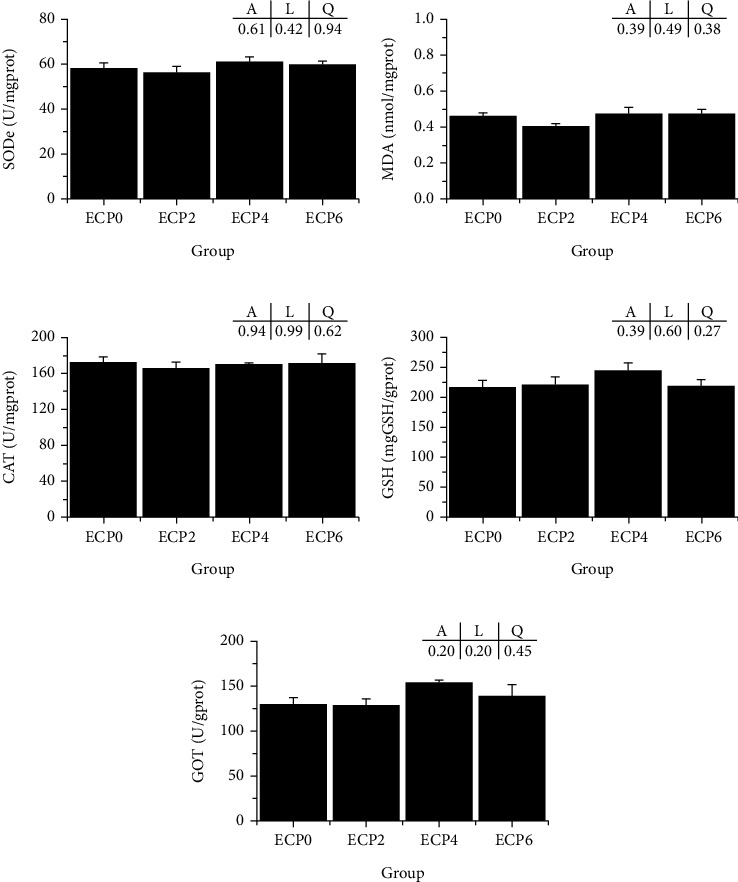
Effects of different levels of enzymatic cottonseed protein (ECP) on liver antioxidant of Chinese soft-shelled turtle. (a) SOD: superoxide dismutase; (b) MDA: malonaldehyde; (c) CAT: catalase; (d) GSH: glutathione; (e) GOT: glutamic-oxalacetic transaminase; error bars represented the mean ± SE (*n* = 6). *A* is the variance analyzed by one-way ANOVA; *L* is the linear trend analyzed by orthogonal polynomial contrasts; *Q* is the quadratic trend analyzed by orthogonal polynomial contrasts.

Compared with the control group, there was no significant difference in SOD, MDA, CAT, GSH, and GOT in the liver of turtles (*P* > 0.05) (Figure 2).

### 3.4. Intestinal Digestive Enzyme Activity

**Figure 3:**
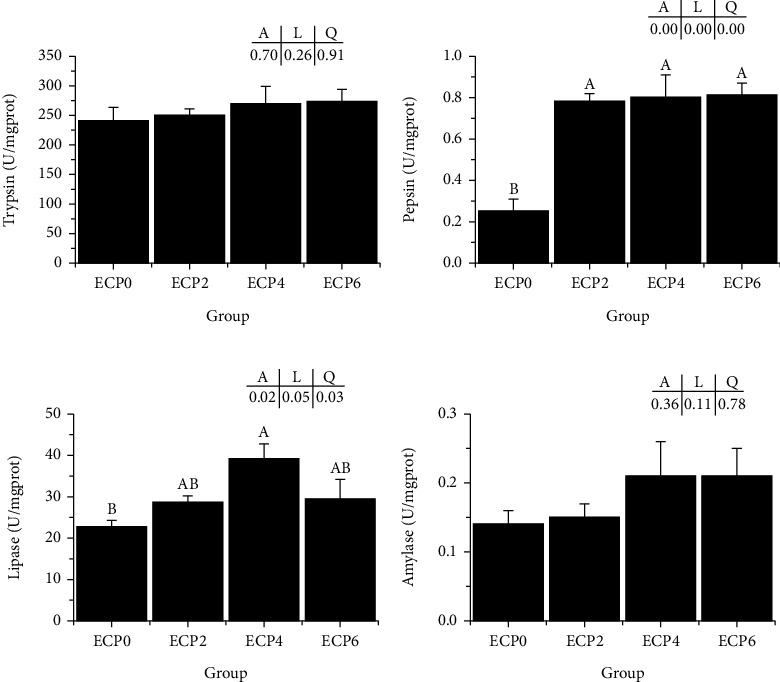
Effects of different levels of enzymatic cottonseed protein (ECP) on intestinal digestive enzyme activity of Chinese soft-shelled turtle. (a) Trypsin; (b) pepsin; (c) lipase; (d) amylase; error bars represented the mean ± standard error (*n* = 6). Different letters indicated significant differences between groups (*P* < 0.05). *A* is the variance analyzed by one-way ANOVA; *L* is the linear trend analyzed by orthogonal polynomial contrasts; *Q* is the quadratic trend analyzed by orthogonal polynomial contrasts.

Compared with the control group, all PBM dietary groups significantly increased the activity of intestinal pepsin (*P* < 0.05), and the EPC4 group significantly increased the activity of intestinal lipase (*P* < 0.05). However, there were no significant differences in trypsin and amylase activities among the groups (*P* < 0.05) (Figure 3).

### 3.5. Histological Study of the Intestine

**Figure 4:**
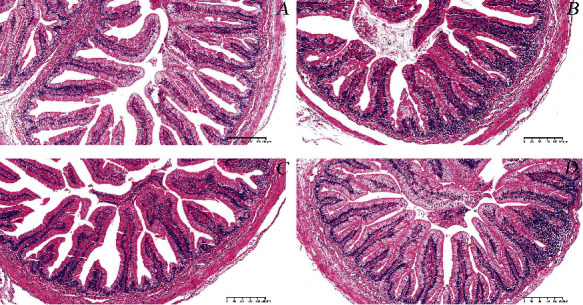
Histological sections of the intestines of a Chinese soft-shelled turtle. Magnification times are 100x. (a) Turtle fed with Con diet. (b) Turtle fed with ECP2 diet. (c) Turtle fed with ECP4 diet. (d) Turtle fed with ECP6 diet. The scale bar was 200 *μ*m.

Compared with the control group, the intestinal villi height of the EPC4 and EPC6 groups was significantly increased (*P* < 0.05), the villi width of the EPC2 and EPC4 groups was significantly increased (*P* < 0.05), and the intestinal muscle layer thickness of EPC4 group was significantly increased (*P* < 0.05) (Table 5, Figure 4).

### 3.6. Intestinal Inflammation-Related Gene Expression

**Figure 5:**
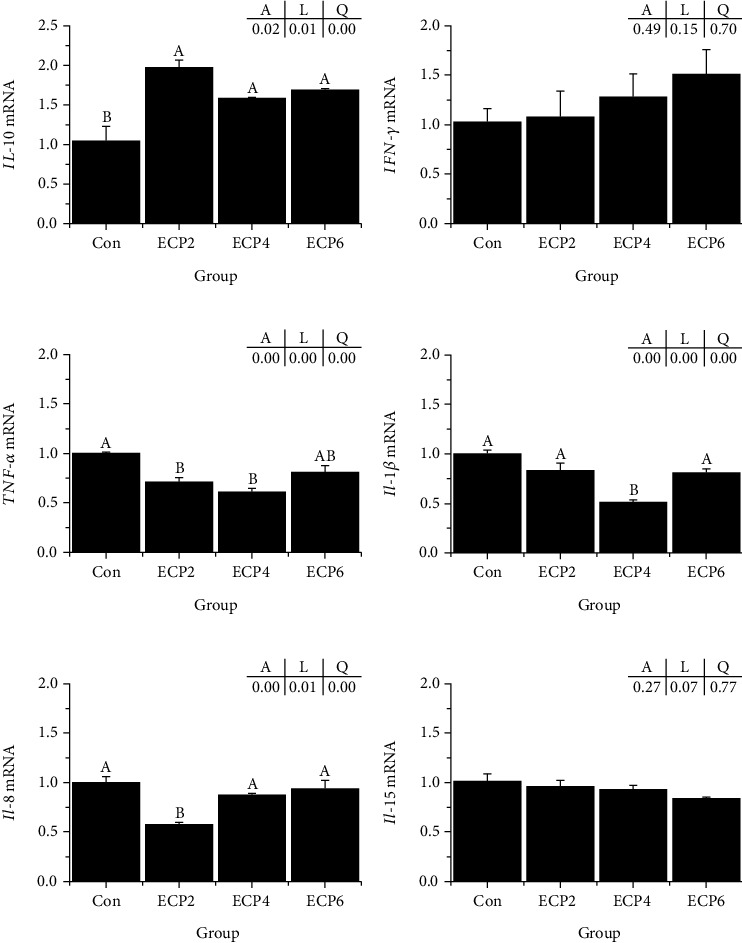
Effects of different levels of enzymatic cottonseed protein (ECP) on intestinal inflammation-related genes of Chinese soft-shelled turtle. Error bars represented the mean ± standard error (*n* = 6). Different letters indicated significant differences between groups (*P* < 0.05). *A* is the variance analyzed by one-way ANOVA; *L* is the linear trend analyzed by orthogonal polynomial contrasts; *Q* is the quadratic trend analyzed by orthogonal polynomial contrasts.

There was no significant difference in the gene expression of IFN*-γ* and IL-15 among the groups (*P* > 0.05). Compared with the ECP0 group, the expression of the IL-10 gene in the experimental group was significantly upregulated (*P* < 0.05), the gene expression of *TNF-α* and IL-8 in the ECP2 group was significantly downregulated (*P* < 0.05), and the expression of TNF-*α*, IL-8 in the ECP4 group was significantly upregulated (*P* < 0.05). The expression of the IL-1*β* gene was significantly downregulated (*P* < 0.05) (Figure 5).

## 4. Discussion

The free amino acids and small peptides released after the enzymatic of plant protein promote growth and intestinal absorption [34]. Previous studies have found that the addition of enzymatic protein products to the basal diet increased the growth of crucian carp (*Carassius auratus)* [16], channel catfish (*Ictalurus punctatus*) [35], and ricefield eel (*Monopterus albus*) [36]. This experiment showed that the replacement of fish meal with enzymatic cottonseed protein demonstrated good growth performance in Chinese soft-shelled turtles. These results indicated that the replacement of fish meal with enzymatic cottonseed protein showed positive effects on the growth performance of Chinese soft-shelled turtles. The reason why enzymatic cottonseed protein can effectively replace fish meal is that enzymatic cottonseed protein contains more easily absorbed small peptides and amino acids and other nutrients [37]. Rich, small peptides can stimulate the secretion and activity of digestive enzymes [38], promoting the development of intestinal mucosal structure and function which improves the intestinal absorption of nutrients [22]. However, it is worth noting that continuing to add a high proportion of enzymatic cottonseed protein may not further improve the growth of turtle, which was consistent with previous studies on largemouth bass (*Micropterus salmoides*) [39] and turbot (*Scophthalmus maximus*) [40]. In this study, there was no significant difference in whole-body composition between groups, indicating that enzymatic hydrolysis of cottonseed protein had little effect on the whole-body composition of Chinese soft-shelled turtle. The current study also is in line with other studies that illustrated that the replacement of fish meal with enzymatically hydrolyzed cottonseed protein did not affect the whole-body composition of blunt snout bream (*Megalobrama amblycephala*) [41] and largemouth bass [42]. However, studies have shown that enzymatically cottonseed protein in feed affects the whole-body composition of largemouth bass and turtle [43, 44]. It is hypothesized that the contradicting findings may be due to differences in cottonseed protein production methods and enzymatic hydrolysis processes [45].

An increase in total protein content in serum represents an increase in the body's immune capacity [46], and globulin is the main part of serum proteins and plays a key role in immune responses [47]. In this experiment, the total protein and globulin increased with the increase of enzymatic cottonseed protein in the diet and reached the maximum in the ECP4 group. The results indicated that adding an appropriate proportion of enzymatic cottonseed protein to the diet improved the nonspecific immunity of turtles. Song et al. [48] found that adding enzymatic protein to the diet can improve sea cucumbers' immunity and disease resistance. Li et al. [49] found that adding enzymatic soybean meal to the diet can improve the immunity of largemouth bass. After the protein is enzymatically hydrolyzed, bioactive peptides with immune stimulation can be produced, which positively enhances the nonspecific immunity of aquatic animals [50].

The body's antioxidant defense system can protect cells and cell membranes from oxidative damage and maintain normal physiological functions of the body [51]. The antioxidant capacity of fish is affected by nutritional factors [52]. SOD, CAT, and GSH are important antioxidants in aquatic animals, and their activity and content reflect the antioxidant capacity [53]. MDA is a product of body peroxidation, and its content reflects tissue damage [54]. In this experiment, there was no significant difference in liver SOD, CAT, GSH, or MDA between the experimental groups and the control group, indicating that enzymatic cottonseed protein replacing fish meal had no adverse effect on the antioxidant capacity of the Chinese soft-shelled turtle. Studies have shown that enzymatic hydrolysis of cottonseed protein improves the antioxidant capacity of largemouth bass [42], and enzyme-treated plant protein has a more influential role in improving antioxidant capacity [55]. It is hypothesized that these findings are inconsistent with this study, possibly due to different aquatic animals.

The activity of intestinal digestive enzymes reflects the ability of aquatic animals to digest and absorb nutrients entering the body and then determines the growth rate of aquatic animals [56]. In this experiment, ECP replacing fish meal significantly increased intestinal pepsin activity, and 4% ECP replacing fish meal significantly increased intestinal lipase activity. This is consistent with the results of studies on turbot [57], pacific white shrimp (*Litopenaeus vannamei*) [58], and ricefield eel [36]. These results show that the inclusion of enzymatically cottonseed protein in the diet can improve the digestive ability of turtles possibly due to the rich small peptides and free amino [59].

The intestine is an important organ for the digestion and absorption of aquatic animals, and its digestion and absorption capacity are closely related to the biochemical barrier function [60]. The length and width of the intestinal villi and the thickness of the muscular layer are important indicators for measuring the digestion and absorption capacity of aquatic animals [61]. The increase in the length and width of the intestinal villi can increase the contact area with food, and the thickness of the muscular layer can increase the reflected intestinal contractility [44], thereby promoting digestion [62]. The present study showed that the addition of enzymatic cottonseed protein in the diet increased the length and width of the villi and the thickness of the muscle layer, which was beneficial to the absorption of nutrients. Moreover, other studies have demonstrated similar results in Nile tilapia (*Oreochromis niloticus*) [63] and channel catfish [64]. The main reason for these findings may be that small peptides can promote the development of intestinal mucosal structure and enhance the absorption capacity of the intestine [65].

The intestine has the functions of digesting food and absorbing nutrients, and it is a vital immune organ of aquatic animals [66]. The surface of the intestinal tract is in extensive contact with a large number of bacteria, viruses, biological toxins, and chemical toxins [67]. Intestinal mucosal tissue has become the most threatened part of the body [68], which can easily induce intestinal inflammation, resulting in various intestinal diseases [69]. Cytokines related to inflammation in aquatic animals mainly include proinflammatory factors (TNF-*α*, IL-1*β*, etc.) and anti-inflammatory factors (TGF-*β*, IL-10, etc.) [70]. Mengya et al. [71] showed that adding cottonseed protein to the diet of largemouth bass increased the risk of inflammation. Li et al. [72] showed that adding soybean meal to a turbot diet caused intestinal damage. In this study, different proportions of ECP replacing fish meal could upregulate the expression of the IL-10 gene, the expressions of TNF-*α* and IL-8 genes were significantly downregulated when ECP replaced 2% fish meal, and TNF*-α* and IL-1*β* genes' expression was significantly downregulated when ECP replaced 4% fish meal. Zhang et al. [42] added ECP to the diet of largemouth bass, which also inhibited the expression of inflammatory factors. Other studies have shown that enzymatically treated protein sources can prevent intestinal inflammation in aquatic animals [73, 74]. The reason may be that the amino acids and small peptides of cottonseed protein increase after enzymatic, which helps aquatic animals improve animal immunity.

## 5. Conclusions

In summary, this study showed that enzymatic cottonseed protein replacing fish meal positively affected the growth performance, immunity, and digestion of Chinese soft-shelled turtles. Besides that, it was noted that dietary treatment of enzymatic cottonseed protein prevents intestinal inflammation and promotes the immunity and intestinal health of Chinese soft-shelled turtles. Therefore, the recommended replacement of enzymatic cottonseed protein with a fish meal in Chinese soft-shelled turtle feed is 4%.

## Figures and Tables

**Table 1:** Ingredients and chemical composition of the experimental diets (%).

Ingredients	Dietary treatment^1^		
	ECP0	ECP2	ECP4	ECP6	
Fish meal^2^	55.00	53.00	51.00	49.00	
Enzymatic cottonseed protein^3^	0.00	2.00	4.00	6.00	
Soy protein concentrate	5.00	5.00	5.00	5.00	
Wheat meal	25.80	25.64	25.48	25.32	
Soybean oil	3.00	3.16	3.32	3.48	
Extruded soybean	5.00	5.00	5.00	5.00	
Shrimp paste	3.00	3.00	3.00	3.00	
CaH_2_PO_4_	2.00	2.00	2.00	2.00	
Choline chloride	0.20	0.20	0.20	0.20	
Vitamin premix^4^	0.50	0.50	0.50	0.50	
Mineral premix^5^	0.50	0.50	0.50	0.50	

Proximate composition (% dry matter)			
Crude protein	43.89	43.77	43.65	43.54
Crude lipid	8.46	8.46	8.45	8.45
Ash	14.61	14.49	14.37	14.25

^1^ECP0, ECP2, ECP4, and ECP6 (no replacement of fish meal, ECP replacement of 2%, 4%, 6% of fish meal). The same as below. ^2^Fish meal, crude protein 63.73%, and crude lipid 6.67%. ^3^Enzymatic cottonseed protein, crude protein 62.00%, and crude lipid 0.40% (Chengdu Meiyide Biotechnology Co., Ltd). ^4^Vitamin premix: provided by Zhejiang Jindadi Agricultural Co., Ltd., Zhejiang, China. ^5^Mineral premix: provided by Zhejiang Jindadi Agricultural Co., Ltd., Zhejiang, China.

**Table 2:** Primers sequence of intestinal inflammation-related genes in Chinese soft-shelled turtle.

Gene	Accession number	Pair primers	Size (bp)	Tm (°C)
*β-*Actin	XM_006134860	F:TGAGCTTCGTGTAGCACCTG	252	58.18
		R:AGGATGGCATGGGGTAAAGC		58.02
IL-10	KT203380	F:ACAGGAAATATGGGGAAGGACG	126	56.79
		R:AAGATTTAAACTGAGGTTCTGGAAG		52.13
IFN-*γ*	JN021380	F:GTCCCAACCAACGGCAAAC	195	57.87
		R:GACTTTGTTGCTTCAAACGGG		54.85
TNF-*α*	XM_014575959	F:TCCTCCGGCACATCATCTTG	116	57.51
		R:GTACCACACTTCGGTCTCGG		58.18
IL-1*β*	NM_001317048	F:TCCAACACCAAGTGAGGCTG	249	57.90
		R:ACTCAAACTGGGTGGTGTCC		57.64
IL-8	FJ472848	F:AGTGAGTTGTTTGTCCTCAATTAGT	209	53.47
		R:AGCTTGGGGCAGAGAAGAGA		58.53
IL-15	XM_006121622	F:ACATACGTGAAGATGAATGTGAAGT	116	53.51
		R:GCGCACATGCTGTTGGATTT		57.07

*β*-Actin: beta-actin; IL-10: interleukin-10; IFN-*γ*: interferon-*γ*; TNF-*α*: tumor necrosis factor-*α*; IL-1*β*: interleukin-1*β*; IL-8: interleukin-8; IL-15: interleukin-15.

**Table 3:** Effects of different levels of enzymatic cottonseed protein (ECP) on growth performance of Chinese soft-shelled turtle (mean ± SE, *n* = 4).

	Dietary treatment				*Pr* > *F*^1^		
	ECP0	ECP2	ECP4	ECP6	ANOVA	Linear trend	Quadratic trend
IBW (g)	3.44 ± 0.00	3.44 ± 0.00	3.44 ± 0.00	3.44 ± 0.00			
FBW (g)	30.88 ± 0.19^b^	33.76 ± 0.74^a^	33.84 ± 0.46^a^	33.15 ± 0.66^ab^	0.01	0.02	0.01
SR (%)	87.50 ± 4.17	92.50 ± 2.50	91.67 ± 5.18	93.33 ± 3.04	0.65	0.28	0.75
WG (%)	798.02 ± 5.40^b^	880.25 ± 21.72^a^	882.92 ± 13.17^a^	863.69 ± 19.25^ab^	0.01	0.02	0.01
FCR	0.88 ± 0.03^a^	0.76 ± 0.02^b^	0.77 ± 0.01^b^	0.79 ± 0.00^b^	0.00	0.01	0.00
SGR (%/d)	3.92 ± 0.01^b^	4.07 ± 0.04^a^	4.08 ± 0.02^a^	4.04 ± 0.04^a^	0.01	0.01	0.01

IBW: initial mean body weight; FBW: final mean body weight; WG: weight gain; SR: survival rate; FCR: feed conversion rate; SGR: specific growth rate. Mean values in the same row with different superscripts are significantly different (*P* < 0.05). ^1^Significance probability associated with the *F*-statistic.

**Table 4:** Effects of different levels of enzymatic cottonseed protein (ECP) on whole-body proximate composition of Chinese soft-shelled turtle (wet basis, mean ± SE, *n* = 4).

	Dietary treatment				*Pr* > *F*^1^		
	ECP0	ECP2	ECP4	ECP6	ANOVA	Linear trend	Quadratic trend
Moisture (%)	69.46 ± 1.85	71.26 ± 0.74	68.54 ± 1.26	70.53 ± 0.35	0.45	0.93	0.94
Crude protein (%)	19.93 ± 1.35	18.49 ± 0.57	20.01 ± 1.01	18.51 ± 0.26	0.52	0.52	0.98
Crude lipid (%)	3.99 ± 0.39	3.42 ± 0.18	4.40 ± 0.33	3.91 ± 0.09	0.21	0.57	0.89
Ash (%)	4.94 ± 0.27	4.68 ± 0.35	5.13 ± 0.28	5.04 ± 0.18	0.71	0.54	0.75

^1^Significance probability associated with the *F*-statistic.

**Table 5:** Effects of different levels of enzymatic cottonseed protein (ECP) on intestinal villus height, villus width, and muscle thickness of Chinese soft-shelled turtle.

	Dietary treatment				*Pr* > *F*^1^		
	ECP0	ECP2	ECP4	ECP6	ANOVA	Linear trend	Quadratic trend
Villus height (*μ*m)	336.69 ± 20.22^b^	389.31 ± 14.32^ab^	422.52 ± 14.45^a^	408.16 ± 6.71^a^	0.00	0.00	0.03
Villus width (*μ*m)	107.41 ± 7.19^c^	136.46 ± 1.65^a^	130.96 ± 5.37^ab^	114.46 ± 1.37^bc^	0.00	0.46	0.00
Muscle layer thickness (*μ*m)	60.95 ± 1.05^b^	62.95 ± 2.29^ab^	72.07 ± 4.67^a^	60.36 ± 1.49^b^	0.02	0.56	0.02

Note: values are presented as means ± standard error (*n* = 6). Mean values in the same row with different superscripts are significantly different (*P* < 0.05).

## Data Availability

All data to support the findings of this study are included in this paper.
